# Predictive factors of frozen section in transoral microlaryngeal surgery for suspicious glottic lesions

**DOI:** 10.1016/j.bjorl.2024.101434

**Published:** 2024-04-17

**Authors:** Mateus Morais Aires, Fábio Yukio Pereira I, Camilla Diacópulos Silva, José Eduardo de Sá Pedroso, Noemi Grigoletto de Biase, Leonardo Haddad

**Affiliations:** a Universidade Federal de São Paulo (UNIFESP), Departamento de Otorrinolaringologia e Cirurgia de Cabeça e Pescoço, São Paulo, SP, Brazil; b Universidade de Pernambuco (UPE), Faculdade de Ciências Médicas, Recife, PE, Brazil; c Pontifícia Universidade Católica de São Paulo (PUC), São Paulo, SP, Brazil

**Keywords:** Frozen section, Larynx diseases, Neoplasms, Vocal cords, Dysplasia

## Abstract

**Objective:**

Frozen biopsy may guide surgical intraoperative decisions. We evaluated the accuracy of frozen biopsy for diagnosing benign, dysplastic and malignant laryngeal lesions, compared to paraffin section (gold standard).

**Methods:**

Retrospective review of the charts of all patients presenting with laryngeal lesions suspicious of malignancy, who underwent laryngeal microsurgery with frozen biopsy in our institution, between 2015 and 2020. Results of frozen biopsy and paraffin section examinations were compared.

**Results:**

Among 113 samples of 89 patients, paraffin section diagnosed 23 benign, 31 dysplastic and 59 malignant lesions. The accuracy of the frozen biopsy in identifying dysplasia or malignancy was 80.5% (91/113), and greater for lesions >5 mm (78.8% × 51.5%; *p* = 0.009). The positive and negative predictive values, sensitivity and specificity were 95.9%, 51.3%, 78.9% and 86.9%, respectively.

**Conclusions:**

Frozen section is a reliable tool when malignancy is detected, but almost half of benign results exhibit dysplasia or malignancy in paraffin section. Other clinical parameters should be considered in intraoperative decisions to prevent undertreatment.

**Level of evidence:**

4.

## Highlights

• Frozen biopsy isolated is not a reliable tool for guiding surgical decisions when it comes negative.
• Frozen biopsy is reliable for malignant results.
• Larger biopsy fragments are associated with a more reliable diagnosis.

## Introduction

Smaller laryngeal lesions suspicious of malignancy often require microsurgery to acquire material for histopathological analysis.^1^, ^2^ Treatment is carried out according to the nature of the lesion. Benign lesions may only demand clinical follow-up. For dysplasias, which are premalignant lesions, the treatment involves complete excision of the lesion, with a minimum margin (excisional biopsy).^3^, ^4^ In the case of laryngeal carcinomas, oncological surgery is performed – either cordectomies or open laryngectomies – for the excision of the tumor with margins of at least 2 mm for early-stage lesions.^5^ In some cases of severe dysplasia or early laryngeal carcinoma, especially in extensive superficial lesions, radiotherapy may also be an option.^1^, ^6^

The gold standard method for histopathological analysis remains the paraffin-embedded tissue section.^7^ However, a frozen section biopsy has the advantage of providing a quick, intraoperative result, which is able to guide the surgeon’s decisions during the procedure.^8^ It plays an important role in the treatment of head and neck cancer. Among the determinations made by frozen section analysis include the definition of the nature and histological type of a suspicious lesion, evaluation of adequacy of surgical margins of resection, and evaluation of lymph nodes for the presence of metastatic disease.^9^

The concordance between frozen and paraffin sections seems to depend on the surgeon’s precision in choosing the fragment to be assessed, experience of the pathologist, and technical artifacts such as cautery, crush, or freeze artifacts.^5^, ^10^

Frozen section biopsy may be decisive in the surgeon’s intraoperative approach to a laryngeal suspicious lesion. In the case of a benign result, it enables saving healthy tissue from the vocal fold, with a greater chance of preserving vocal quality. Faced with a result of dysplasia or malignancy, it immediately enables adequate resection. In these cases, it may save the patient from undergoing a further second surgical procedure to complete the treatment, thereby avoiding the additional anesthetic risk and the psychological repercussions on both patients and their families. In addition, the frozen section biopsy is cost-effective since it adds a cost of just 58 Euros to the surgical procedure, while further hospitalization, general anesthesia, and surgery would cost 1064 Euros to healthcare systems.^5^

This study aimed to assess the accuracy of frozen section biopsy in cases of laryngeal lesions suspected of malignancy, with respect to diagnosing benign, dysplastic, and malignant lesions. The assessment involved a comparison with paraffin section analysis, determining sensitivity, specificity, Negative Predictive Value (NPV) and Positive Predictive Value (PPV) of frozen section.

## Methods

Patients presenting with a glottic lesion suspicious of malignancy (leukoplakia, leukoerythroplakia, and exophytic lesions), who underwent microsurgery with intraoperative frozen biopsy, were included. The medical charts of all consecutive patients treated in our department between 2015 and 2020 were reviewed. Patients with incomplete medical records were excluded. We obtained the approval of the ethics committee in our institution to perform this study (number: 4528743). Informed consent forms were signed by all patients participating in the study. This study was conducted in accordance with the 2002 World Medical Association Declaration of Helsinki.

A retrospective analysis of the medical records was performed, which included demographic data, information on the performed surgery, and the results of the frozen section biopsy and of the histopathological analysis using paraffin section. According to the pathology routine, the same fragment studied in the frozen section was later sent for paraffin section analysis, and the results were compared.

### Surgical technique and histopathological analysis

All specimens were obtained through laryngeal microsurgery, performed by the senior authors (JESP and LH), using cold instruments, electrocautery, or CO_2_ laser. Whenever possible, a type I cordectomy with cold steel was performed, preserving the vocal ligament, and the entire piece was sent for frozen section analysis. If the result was malignant, the resection was extended to an oncological surgery with adequate margin, at least a type II cordectomy. In the case of infiltrative lesions with high suspicious features and/or with invasion of the vocal ligament, a type II cordectomy was initially performed, and the entire piece was sent for frozen section analysis. Types II to VI cordectomies were performed with either electrocautery or CO_2_ laser. When complete excision was not possible, as in diffuse bilateral lesions, or if the patient had previously opted for radiotherapy on a malignant result, an incisional biopsy was executed, and the most suspicious area was selected for frozen biopsy. Both the frozen sections and the paraffin sections were stained with Hematoxylin-Eosin.

Various terminologies were used in the histopathological report of the frozen biopsy. To assess the concordance with paraffin section, these terms were classified into 3 groups of results: benign (benignity, acanthosis, keratosis, parakeratosis, nonspecific inflammatory infiltrate, chronic granulomatous inflammatory process), dysplasia (dysplastic epithelium, mild dysplasia, moderate dysplasia, severe dysplasia, carcinoma in situ) and malignant (microinvasive and invasive carcinoma). Dysplasia was further categorized according to the 2017 World Health Organization (WHO) classification, in which squamous hyperplasia and mild dysplasia are considered to be low-grade dysplasia, while moderate dysplasia, severe dysplasia and carcinoma in situ are considered to be high-grade dysplasia.^11^, ^12^ For some patients, there were multiple samples, and all the samples were considered.

Overdiagnosis was defined when the frozen biopsy result was overestimated in comparison to the paraffin section (for example, malignancy in the frozen biopsy and dysplasia in the paraffin section). Underdiagnosis was defined when the frozen biopsy result was underestimated in comparison to the paraffin section (for example, benignity in the frozen biopsy and dysplasia in the paraffin section). The samples sent for frozen biopsy were classified according to their size into smaller or larger than 5 millimeters (mm).

### Statistical analysis

Statistical analysis was performed using SPSS 23.0 for Windows (SPSS Inc., Chicago, IL, USA). Categorical data were expressed as N and percentages and analyzed using the Chi-Square test. Positive and negative predictive values, sensitivity, specificity, and accuracy were calculated from applying the Bayes’ theorem, considering the result of the paraffin section as the gold standard. All statistical evaluations were two-tailed, with a significance level defined as *p* = 0.05.

## Results

Eighty-nine patients met the inclusion criteria, totaling 113 laryngeal samples. The average age was 64.4 years, ranging from 26 to 102 years. Most patients were male (n = 69; 77.5%) and presented current or previous history of smoking (n = 74; 83.1%). The average time to perform the frozen section biopsy was 21 minutes. The mean follow-up period was 37.5 months and ranged from 6 to 72 months.

The lesions were classified as leukoplakia (n = 26; 29.2%), leukoerythroplakia (n = 19; 21.3%) and exophytic (n = 44; 49.4%). The instruments used in the laryngeal microsurgery to obtain the samples were cold steel (n = 37; 32.7%), electrocautery (n = 34; 30.0%) and CO_2_ laser (n = 42; 37.2%). The surgeries performed were incisional biopsy (n = 29; 25.7%), type I (n = 24; 21.2%), type II (n = 16; 14.2%), type III (n = 30; 26.5%), and types IV to VI (n = 14; 12.4%) cordectomies (Table 1).

**Table 1:** Classification of the lesion aspect, instrument, and surgical technique.

Classification	Nº of samples	%
Lesion aspect		
Leukoplakia	26	29.2
Leukoerythroplakia	19	21.3
Exophytic	44	49.4
Instrument		
Cold steel	37	32.7
Electrocautery	34	30.0
CO_2_ laser	42	37.2
Surgical technique		
Incisional biopsy	29	25.7
Type I cordectomy	24	21.2
Type II cordectomy	16	14.2
Type III cordectomy	30	26.5
Types IV‒VI cordectomy	14	12.4

Nº, Number.

Paraffin section diagnosed 23 (20.3%) benign, 31 (27.4%) dysplastic and 59 (52.2%) malignant lesions among the 113 samples included in this study. The concordance of the frozen biopsy with paraffin section for benign, dysplastic, and malignant lesions was 51.3% (20/39), 41.0% (16/39) and 94.3% (33/35), respectively (Table 2). Among 59 malignant lesions diagnosed with paraffin section, 33 (55.9%) were correctly identified by the frozen biopsy.

**Table 2:** Comparison of the frozen biopsy with paraffin section.

Frozen biopsy result	Nº of samples	Nº of correct diagnoses (%)	Nº of underdiagnoses (%)	Nº of overdiagnoses (%)
Benign	39	20 (51.3%)	19 (48.7%)	N/A
			Dysplasia: 13 (33.3%)	
			Malignant: 6 (15.4%)	
Dysplasia	39	16 (41.0%)	20 (51.3%)	3 (7.7%)
Malignant	35	33 (94.3%)	N/A	2 (5.7%)
				Dysplasia: 2 (5.7%)
Total	113	69 (61.1%)	39 (34.5%)	5 (4.4%)

Nº, Number; N/A, Not Applicable.

Analyzing the frozen biopsy results of dysplasia, 20 were low-grade and 19 were high-grade. Six out of 20 (30.0%) low-grade dysplasias were underdiagnosed and were malignant in the paraffin section. Fourteen out of 19 (73.7%) high-grade dysplasias were underdiagnosed and were malignant in the paraffin section.

To assess the reliability of the frozen biopsy to guide the intraoperative decision of the surgeon, the results were grouped into (1) Dysplastic or malignant – which demand complete resection -, and (2) Benign – which do not demand resection. The accuracy of the frozen biopsy in identifying a lesion that demands resection (group 1) was 80.5% (91/113). A result of “dysplasia” or “malignancy” presented a positive predictive value of 95.9%, a negative predictive value of 51.3%, sensitivity of 78.9%, and specificity of 86.9%, in the differentiation from a benign lesion (Table 3).

**Table 3:** Comparison between the frozen biopsy and paraffin section, grouping dysplasias and malignant lesions, for evaluation of accuracy, sensitivity, specificity, and positive and negative predictive values.

		Nº of paraffin section samples		
		Dysplasia or Malignant	Benign	Total
Nº of frozen biopsy samples	Dysplasia or Malignant	71	3	74
	Benign	19	20	39
Total		90	23	

Nº, Number.

The frozen biopsy samples were classified according to size: 33 (33.3%) were >5 mm and 66 (66.7%) were ≤5 mm. Fourteen samples had no description of the size and were excluded from this analysis. The accuracy was greater for lesions >5 mm (78.8% × 51.5%; *p* = 0.009).

We analyzed the concordance between frozen and paraffin sections according to the instrument used. It was slightly higher when using CO_2_ laser (69.0%), comparing to cold steel (59.4%) and electrocautery (52.9%), but there was no statistical significance (Chi-Square test, *p* = 0.35; Table 4). According to the surgical technique, the frozen section accuracy was higher in types IV, V and VI cordectomies, when comparing to incisional biopsy and types I to III cordectomies, but there was also no statistical significance (Chi-Square test, *p* = 0.37; Table 5).

**Table 4:** Comparison of the frozen biopsy with paraffin section, according to the surgical instrument.

Instrument	Nº of samples	Nº of correct diagnoses (%)	Nº of incorrect diagnoses (%)
Cold steel	37	22 (59%)	15 (41%)
Electrocautery	34	18 (53%)	16 (47%)
CO_2_ laser	42	29 (69%)	13 (31%)

Nº, Number. Chi-Square test, *X*^2^ = 2.1, *p* = 0.35.

**Table 5:** Comparison of the frozen biopsy with paraffin section, according to the surgical technique.

Technique	Nº of samples	Nº of correct diagnoses (%)	Nº of incorrect diagnoses (%)
Incisional biopsy	29	17 (59%)	12 (41%)
Type I cordectomy	24	13 (54%)	11 (46%)
Type II cordectomy	16	9 (56%)	7 (44%)
Type III cordectomy	30	18 (60%)	12 (40%)
Types IV, V and VI cordectomies	14	12 (86%)	2 (14%)
Total	**113**	**69 (61.1%)**	**44 (39.9%)**

Nº, Number. Chi-Square test, *X*^2^ = 4.3, *p* = 0.36.

## Discussion

The treatment of a suspicious laryngeal lesion varies according to the finding of benignity, dysplasia, or malignancy in the histopathological analysis. Our aim was to assess the concordance of the intraoperative frozen biopsy with routine postoperative paraffin section, thereby estimating its reliability in guiding the surgeon’s intraoperative conduct.

For a “malignant” result in the frozen biopsy, the high PPV of 95.9% becomes reliable to propose resection with oncological margins. This data is consistent with previous studies. In a paper with 97 patients presenting with early laryngeal carcinomas, Remacle et al. reported accuracy of 94.8% of the frozen biopsy for diagnosing malignancy.^5^ In this assessment, however, the authors considered severe dysplasia as a malignant result and did not analyze other degrees of dysplasia. In a cohort of 68 patients with suspicious laryngeal lesions, Cikojević et al. described an accuracy of 95.7% of the frozen biopsy for diagnosing malignancy.^13^ But many dysplastic lesions were considered as benign even with the knowledge of differences in treatment and prognosis.^3^

On the other hand, the NPV of the frozen biopsy was 51.3%. It means that almost half of these lesions classified as “benign” were underdiagnosed, and 15.4% of the benign results in the frozen biopsy were invasive carcinomas. Similarly, for a “dysplasia” result in the frozen biopsy, the accuracy was only 41.0%, and 51.3% of these lesions were malignant in the paraffin section. In a previous study with 100 laryngeal lesions excised with microsurgery, Jin et al. estimated the accuracy of the frozen biopsy in differentiating into benign, dysplasia, and malignant.^10^ In this situation, the correctness of the diagnosis of dysplasia in the frozen biopsy was only 18%, and 72% of these lesions were invasive carcinomas in the paraffin section.

Grouping the lesions into (1) Dysplastic or malignant – which require total excision of the lesion-, and (2) Benign – which do not require resection of additional tissue-, the frozen biopsy presented a global accuracy of 80.5%. It is, therefore, moderately reliable in guiding the immediate conduct of the surgeon. A result of dysplastic or malignant lesion presents high positive predictive value (95.9%) and specificity (86.9%), thereby proving to be adequate to safely indicate resection of the lesion. However, given the low negative predictive value (51.3%), many “benign” results in frozen biopsy will actually be dysplastic or malignant in paraffin section. In this situation, the frozen biopsy may induce the undertreatment.

These findings reinforce the need for the surgeon to consider other variables to define the treatment. Factors such as patient age, tobacco use, alcohol abuse, extensive, rough, recurrent, and elevated leukoplakia, erythroleukoplakia, reduced mucosal wave in laryngostroboscopy, and transversal vascular pattern in Narrow Band Imaging indicate a greater likelihood of high-grade dysplasia or malignancy. These factors may help to indicate total resection of the lesion, despite a negative result in the frozen biopsy.^1^, ^2^, ^4^, ^14^, ^15^, ^16^

The most prominent factors that may be related to an incorrect diagnosis in the frozen biopsy are the presence of mixed pathologies in the same lesion (one portion with dysplasia and another with invasive carcinoma, for example), superficially performed biopsy (with difficulty in determining the invasiveness), and inadequate sample size.^9^, ^10^, ^17^

Sometimes, the pathologist could bias the result freezing the benign portion of lesion in order to keep other tissues for the paraffin section. In these cases, the fragment studied in the frozen biopsy may not correlate with the malignant lesion, providing an incorrect diagnosis. In our Pathology department, it is routine to freeze the whole piece, without impairing any further analysis of the same specimen in the paraffin section.

Regarding the sample size, we observed greater accuracy for fragments >5 mm. This may be a relevant factor for obtaining an accurate diagnosis with the frozen biopsy. However, some glottic leukoplakias are very thin and small.

The type of instrument and surgical technique could also influence on the frozen section accuracy. To our knowledge, these aspects had not yet been studied in a comparative way. Although we found a slightly greater accuracy when using CO_2_ laser and performing more extensive cordectomies, we did not reach statistical significance. These findings could be due to selection bias, since CO_2_ laser and types IV to VI cordectomies were performed to treat bigger lesions, with higher probability of malignancy. Thus, we cannot conclude that these aspects actually have influence on the frozen section accuracy.

Thus, in view of the global accuracy of the frozen biopsy of 80.5% ‒ with a low negative predictive value of 51% ‒ it is difficult to endorse intraoperative decision-making based exclusively on this exam, when it comes negative. It is suggested that, even if a highly suspicious lesion presents as “benign” in the frozen biopsy, the lesion should be treated as malignant. On the other hand, a “malignant” result in the frozen biopsy is reliable for the surgeon to resect with oncological margin. Efforts have been made to study other predictive biomarkers of malignancy, which, in combination with clinical features, may increase the reliability in distinguishing laryngeal diseases preoperatively.^18^

Our study has some limitations. Although the study was delineated in a tertiary center, with a team of experienced pathologists, no specific training for the study was undertaken. We observed, as described in the literature, a lack of standardization of the histopathological reports, requiring retrospective classification of results by the authors, within the categories proposed by WHO.^11^, ^19^, ^20^ We believe that, in a prospective study, with the possibility of training and immediate discussion with the pathologist regarding the classification of the result, these biases would be minimized. A second limitation is the variation in the method used for biopsy. Although all samples were collected through laryngeal microsurgery, we used cold instruments, electrocautery, and CO_2_ laser, which could alter the global accuracy of the frozen biopsy.

## Conclusions

The frozen section analysis is a reliable tool when malignancy is detected, exhibiting a high positive predictive value of 96%. However, its low negative predictive value of 51% raises concerns, as it has the potential to overlook nearly half of dysplastic or malignant lesions, incorrectly categorizing them as benign. Notably, suspicious lesions larger than 5 mm show a higher accuracy in distinguishing between benign and malignant lesions precisely. Neither the instrument nor the surgical technique influence on frozen section reliability.

## Funding

The authors have no funding or financial relationships to disclose.

## Conflicts of interest

The authors declare no conflicts of interest.
